# Prospective use of amniotic mesenchymal stem cell metabolite products for tissue regeneration

**DOI:** 10.1186/s13036-023-00331-1

**Published:** 2023-02-09

**Authors:** Andang Miatmoko, Berlian Sarasitha Hariawan, Devy Maulidya Cahyani, Syarifah Sutra Dewangga, Kevin Ksatria Handoko, Purwati, Ram Kumar Sahu, Dewi Melani Hariyadi

**Affiliations:** 1 Department of Pharmaceutical Science, Faculty of Pharmacy, Universitas Airlangga, Nanizar Zaman Joenoes Building, Campus C UNAIR Mulyorejo, Mulyorejo, Surabaya, East Java 60115 Indonesia; 2 Stem Cell Research and Development Center, Universitas Airlangga, 2nd Floor Institute of Tropical Disease Building, Campus C UNAIR Mulyorejo, Surabaya, 60115 Indonesia; 3 Master Program of Pharmaceutical Sciences, Faculty of Pharmacy, Universitas Airlangga, Campus C UNAIR Mulyorejo, Surabaya, 60115 Indonesia; 4 Faculty of Pharmacy, Study Program of Pharmacy, Universitas Airlangga, Campus C UNAIR Mulyorejo, Surabaya, 60115 Indonesia; 5 Department of Pharmaceutical Sciences, Hemvati Nandan Bahuguna Garhwal University (A Central University), Chauras Campus, Tehri Garhwal, Uttarakhand 249161 India

**Keywords:** Quality-adjusted life-year, Amniotic mesenchymal stem cell metabolite products, Growth factors, Tissue injury, Stem cells, Molecular therapy

## Abstract

Chronic disease can cause tissue and organ damage constituting the largest obstacle to therapy which, in turn, reduces patients’ quality-adjusted life-year. Degenerative diseases such as osteoporosis, Alzheimer’s disease, Parkinson’s disease, and infectious conditions such as hepatitis, cause physical injury to organs. Moreover, damage resulting from chronic conditions such as diabetes can also culminate in the loss of organ function. In these cases, organ transplantation constitutes the therapy of choice, despite the associated problems of immunological rejection, potential disease transmission, and high morbidity rates. Tissue regeneration has the potential to heal or replace tissues and organs damaged by age, disease, or trauma, as well as to treat disabilities. Stem cell use represents an unprecedented strategy for these therapies. However, product availability and mass production remain challenges. A novel therapeutic alternative involving amniotic mesenchymal stem cell metabolite products (AMSC-MP) has been developed using metabolites from stem cells which contain cytokines and growth factors. Its potential role in regenerative therapy has recently been explored, enabling broad pharmacological applications including various gastrointestinal, lung, bladder and renal conditions, as well as the treatment of bone wounds, regeneration and skin aging due to its low immunogenicity and anti-inflammatory effects. The various kinds of growth factors present in AMSC-MP, namely bFGF, VEGF, TGF-β, EGF and KGF, have their respective functions and activities. Each growth factor is formed by different proteins resulting in molecules with various physicochemical properties and levels of stability. This knowledge will assist in the manufacture and application of AMSC-MP as a therapeutic agent.

## Background

Degenerative diseases, physical injury to organs, and damage due to chronic conditions such as diabetes can cause organ function loss [1–3]. In the early stages of disease, pharmacological drug therapy is the first-line treatment choice, but it has some drawbacks. For example, there are only five types of drugs for reducing the symptoms of Alzheimer’s disease [4], and pharmacotherapy to slow disease progression is not yet available. For this purpose, therapeutic agents should inhibit extracellular amyloid plaque deposition and intracellular neurofibrillary formation. Additionally, Alzheimer’s therapies that have neuroprotective mechanisms and the use of anti-inflammatory stem cell therapies and the growth factor NDX-1017 are currently under investigation [5, 6].

Stem cells are undifferentiated cells that continuously divide, renew themselves, and differentiate into different types of cells. With the ability of self-renewal, pluripotency, and differentiation, stem cells have great potential for treating various diseases. Stem cells can be divided into two main groups based on their origin: embryonic stem cells and adult stem cells. However, stem cell therapy has two main problems that pose challenges for its therapeutic use. The first relates to availability and mass production. Cell therapy protocols using mesenchymal stem cells (MSCs) for tissue regeneration generally require hundreds of millions of MSCs per treatment. Therefore, expansion of the in vitro cell culture is necessary for longer periods and large bioreactors [7]. The need for cells to replace the disease-induced loss of hepatocytes, pancreatic cells, or cardiomyocytes involves approximately 1 to 10 × 10^9^ functional cells per patient. An even higher requirement has been calculated for the production of “in vitro blood,” as approximately 2.5 × 10^12^ red blood cells are required per patient in transfusion treatments [8]. The main challenge in mass production lies in standardizing the process due to the complexity of pluripotent stem cells and processing cells on a large scale [9].

The use of stem cells can affect the recipient’s immune system. The administered cells can directly induce an immune response or modulate the immune system. This is primarily in the case of cells that are not intended to be used for their essential function (nonhomologous use) or when administered to non-physiological sites, which may change the immunogenicity of the cells. Another risk is bacterial and viral infections. As a cell-based product, stem cell production does not allow for terminal sterilization, purification, virus removal, or inactivation processes. Thus, the risk of transmitting bacterial, viral, fungal, or prion pathogens from the donor to the recipient can lead to life-threatening and even fatal reactions [10].

Because of these limitations, new therapeutic strategies are needed. Stem cell-mediated tissue regeneration involves soluble factors secreted by these cells. Cytokines and growth factors, such as transforming growth factor beta (TGF-β), stromal cell-derived factor 1 (SDF-1), and vascular endothelial growth factor (VEGF), are secreted by stem cells and progenitor cells transplanted into the intestinal space or injected into blood vessels and stimulate many regenerative processes such as neovascularization, activation of tissue intrinsic progenitor cells, decreased apoptosis of endogenous cardiomyocytes, and registration of assistive cells for tissue repair [11, 12]. Additionally, mesenchymal stem cells (MSCs) secrete growth factors and cytokines, which promote wound repair. The combination of growth factors and cytokines successfully induces angiogenesis, reduces inflammation, and promotes fibroblast migration and collagen production [13].

## Amniotic membrane stem cell metabolite products (AMSC-MPs)

### Placental tissue is the primary source of AMSC-MPs

The amnion, chorion, amniotic fluid, and umbilical cord are of fetal origin. These components have been widely studied because of their potential use as cell sources for regenerative therapies. At delivery, the amnionic membrane is strong, protects the fetus from physical shocks, regulates the pH of the fluid membranes, and secretes various cell signals and bioactive molecules as antimicrobials and anti-inflammatory agents [14]. Amniotic membrane stem cells (AMSCs) are MSCs from the amniotic epithelium and the stroma of the amniotic membrane that are sources of epidermal growth factor (EGF) and keratinocyte growth factor (KGF). Furthermore, stem cells synthesize and secrete various extracellular matrix proteins, cytokines, growth factors, and other bioactive proteins that contribute to the healing and regenerative processes [15]. The collection of conditioned stem cell culture media rich in bioactive agents such as growth factor and cytokines secreted into the extracellular space is defined as a metabolite product. Since it is derived from amniotic mesenchymal stem cells, it is referred to as AMSC-MP [7].

The AMSC-MP collection procedures are conducted under sterile conditions. The first step is expanding cells to the desired passage. Then, upon trypsinization and counting of selected cells, the cell needs to be seeded again at a determined cell density, for example, 12,000 cells/cm^2^, and cultured in an appropriate growth medium. After 72 h, the growth medium is removed, and the cells are washed several times with phosphate-buffered saline, preferably without Ca^2+/^Mg^2+^ content, at room temperature. After that, the cell culture medium (pre-warmed to 37 °C) with only 1% antibiotic is added. The AMSC-MP was collected after being incubated for 48 h and centrifuged at 1500 rpm for 3 min two times, then filtered with a syringe filter 0.45 μm to remove debris and dead cells. The AMSC-MP then be dialyzed using a dialysis bag with a proper molecular-weight cut-off to replace the cell growth medium with the phosphate-buffered saline as the media [16, 17].

These molecules include basic fibroblast growth factor (bFGF), EGF, hyaluronic acid (HA), interleukins (IL-1 and IL-10), beta-defensins, TGF-β, elafin, human leukocyte antigen-G, matrix metalloproteinases, tissue inhibitors of metalloproteinases (TIMPs), and platelet-derived growth factor (PDGF) [15]. Additionally, amniotic tissue contains anti-inflammatory factors such as IL-1 and IL-10 receptor antagonists and regulators of catabolic enzymes such as TIMP1, TIMP2, TIMP3, and TIMP4. Furthermore, AMSC-MP is a potent down-regulator of TGF-β signaling, which stimulates the recruitment of fibroblasts and macrophages and upregulates collagen production [18].

### Physicochemical properties and stability of AMSC-MP

AMSC-MP is a clear liquid containing various proteins, and some products form yellowish white lyophilized powder. A study by Kumala et al. (2020) compared the physicochemical stability of AMSC-MP in two forms, i.e., native AMSC-MP liquid and lyophilized AMSC-MP powder [19]. The results showed that AMSC-MP liquid began to change color after 7 days of storage at room temperature. In contrast, AMSC-MP liquid did not show color changes or an odor at cold temperatures. Its pH was 7–7.5 without significant changes during storage for 28 days [19].

**Fig. 1:**
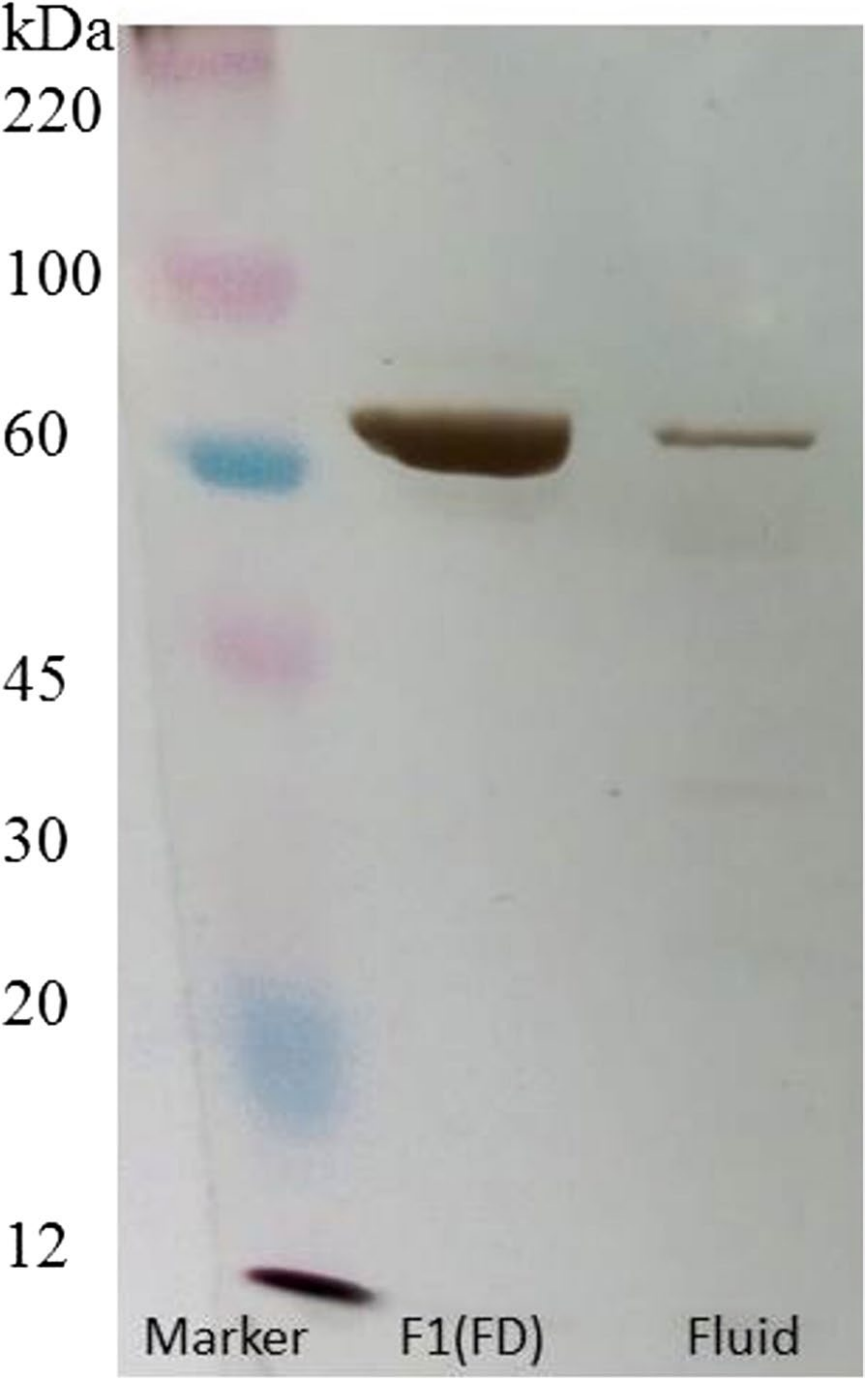
Qualitative determination of protein markers in fluid and freeze-dried (FD) AMSC-MP analysed using SDS-PAGE [19]

AMSC-MP contains several proteins and the major component has a molecular weight of 75.33 kDa, as seen from the thick band in Fig. 1. A stability study measuring TGF-β with the enzyme-linked immunosorbent assay (ELISA) showed that the AMSC-MP liquid is less stable stored in room temperature (25ºC) than when freeze-dried. Freeze-dried AMSC-MP has high crystallinity, which was supported by a scanning electron microscopy characterization showing a tetragonal crystal shape. The crystals have a melting point of 163.8 °C [19].

Specifically, stability data from each component of the AMSC-MP is still limited. It has been reported that the FGF1 and FGF2 lost most of their activity after 6 h at 37 °C [20]. Another study showed that the eye serum drops containing EGF, TGF-β1, and TGF-β2 remained stable in tube segments and vials made from polyvinyl chloride (PVC) with di-2-ethylhexyl phthalate (DEHP) and tris-2-ehtylhexyl trimellitate (TOTM) as the plasticizers, respectively, for about 15 months by storage at -30 °C. However, the stability was significantly decreased by storing samples at 4 °C and 22 °C, which could be reduced up to only 24 and 3 h for EGF and 48 and 9 h for TGF-β1and TGF-β2, respectively. EGF was reportedly unstable at 37 °C [21]. Mizumachi & Ijima (2013) showed that the immobilized-VEGF prepared in a heparin-collagen substrate had higher stability than that of VEGF solution, resulting in about 43% of the initial VEGF retaining its biological activity, although after 16 days of pre-incubation at 37 °C [22]. Meanwhile, the research by Chen & Arakawa (1996) reported that the KGF is unstable in the aqueous media and it follows denaturation and aggregation when heated to 37 °C [23].

## Biochemical components of AMSC-MP

### bFGF

One of the growth factors in AMSC-MP is bFGF [7, 11, 18] (or FGF-2), which is a member of the FGF family that regulates various biological functions including proliferation, morphogenesis, and suppression of apoptosis during development through a complex signal transduction system. bFGF is widely expressed in the nervous system, where it has multiple roles, and it supports the survival and growth of neuronal and neural stem cell cultures [24].

Members of the FGF family have a homologous core region of 120–130 amino acids arranged into 12 antiparallel strands (β1–β12) flanked by divergent amino and carboxyl functional groups. Generally, the sequence variation of the N- and C-terminal ends of specific FGF family members accounts for their differential ligand binding. The heparan sulfate glycosaminoglycan (HSGAG) binding site in the FGF core consists of a β1–β2 loop and parts of β10 and β12. This section differs for each FGF family member and determines the endocrine properties of each. FGF binds to and activates the FGF receptor (FGFR) in the HSGAG-dependent tyrosine kinase receptor family. Upon ligand and HSGAG binding, FGFR dimerizes, allowing the cytoplasmic kinase domain to transphosphorylate and activate tyrosine loop A. Loop phosphorylation is followed by tyrosine phosphorylation in the C tail region, kinase insert, and juxtamembrane region. This process activates the Ras–mitogen-activated protein kinase (MAPK) and phosphoinositide 3-kinase–Akt signaling pathways [25].

In tissue regeneration therapy, FGFs from the paracrine group (FGF1–10, FGF16–18, FGF20, and FGF22) play a role. Paracrine FGFs have a high affinity for HSGAG, activating it and acting locally near the expression source.

### EGF

Another growth factor in AMSC-MP is EGF [11, 12, 17]. EGF is a small polypeptide mitogen present in many species that has been isolated and characterized in breast milk. EGF is a 6-kDa peptide derived from a 1207 amino acid precursor molecule that acts across a 170-kDa membrane glycoprotein receptor. EGF has intrinsic tyrosine kinase activity, like the IGF receptor, and functions in tyrosine kinase-mediated autophosphorylation. TGF-α, which has a 35% amino acid homology with murine EGF and 44% homology with human EGF, also acts through the EGF receptor [26].

Growth factors are naturally produced proteins that regulate cell proliferation, function, and differentiation through receptor signaling. EGF is one of the earliest known polypeptide growth factors and was the founder of the EGF-like family of proteins. EGF is an endogenous peptide that promotes cell growth, proliferation, and differentiation via ligand-receptor (EGFR) interactions [27, 28]. EGF was first isolated from the submaxillary glands of adult male rats by Cohen et al. in 1962. Currently, recombinant human EGF (rhEGF) can be mass produced from *Escherichia coli*, which has accelerated the development of EGF formulations for treating skin conditions such as chronic wounds, burns, diabetic ulcers, and skin aging [28].

EGF has hydrophilic properties [29]. Structurally, EGF is a polypeptide chain with a molecular weight of 6045 Da consisting of 53 amino acids and 6 cysteine residues and has three intramolecular disulfide bonds [27]. EGF is characterized by the absence of three specific amino acid residues, lysine, alanine, and phenylalanine, and it lacks hexosamines and neutral sugars [30]. EGF has an optimal stability at pH 6.0–8.0 and an isoelectric point of about 4.6 [29]. EGF shows poor thermal stability because the protein structure begins to degenerate at 40 °C, and it has a transition midpoint at 55.5 °C. EGF can be completely denatured at temperatures above 76 °C [31].

EGF binds to EGFR; following ligand binding, EGFR (ErbB-1) dimerizes with itself or with a homolog, ErbB-2, ErbB-3, or ErbB-4, increasing intracellular tyrosine kinase activity. This process activates a signaling cascade that has multiple effects: cell proliferation, reduction of apoptosis, and angiogenesis [32].

### TGF-β

TGF-β is an extracellular protein in AMSC-MP produced mainly by a subset of T cells [7, 11, 17]. TGF-β belongs to a group of cytokines collectively referred to as the TGF-β superfamily, whose members regulate epithelial cell growth, differentiation, motility, organization, apoptosis, and tumorigenesis [33]. The TGF-β superfamily consists of a group of polypeptide morphogens. TGFs are divided into two subgroups: the TGF-like subgroup [TGF-βs, activin, nodal, and multiple growth differentiation factors (GDFs)] and the BMP-like subgroup (BMP, GDF, and antimullerian hormone). Members of the TGF-like subgroup exhibit functions in cell adhesion, growth, cytoskeletal organization, survival, proliferation migration, differentiation, chemotaxis, and immune cell activation in multicellular organism development [34].

Blood is the primary source of TGF-β, which promotes healing and tissue regeneration during injury. Platelet aggregation and degranulation release high amounts of TGF-β1 at wound healing sites. Additionally, recruited and activated leukocytes at wound sites secrete various cytokines, including TGF-β1 to support the wound healing process [35]. TGF-β is secreted in a latent or biologically inactive state. During cellular synthesis, the TGF-β precursor undergoes intracellular proteolytic cleavage by furin endopeptidase, resulting in two proteins assembled into dimers via noncovalent associations.

TGF-β signaling involves three parallel pathways, the bone morphogenic protein (BMP), TGF-β, and activin pathways, all of which are major regulators. TGF-β signaling is transduced in cells by several SMAD protein modulators, which eventually enter the cell nucleus and influence the expression of target genes. Since all three pathways comprise ligands and receptors, the combination of different signals allows the regulation of many growth and developmental processes in highly specific ways [36].

### VEGF

VEGF regulates angiogenesis by inducing the proliferation, migration, and permeability of endothelial cells. VEGF is also found in AMSC-MP [7, 11, 17]. During the process of tissue regeneration, VEGF also plays an important role in cardiac repair by decreasing infarction size, reducing remodeling, decreasing endothelial cell apoptosis, supporting angiogenesis and neovascularization, increasing the number of mitotic cardiomyocytes in the border zone, and improving cardiac performance [37]. VEGF is produced by many cell types including tumor cells, macrophages, platelets, keratinocytes, and renal mesangial cells. VEGF activity is not confined to the vascular system; VEGF also plays a role in normal physiological functions such as bone formation, hematopoiesis, and wound healing [38].

The *VEGF* gene is located on chromosome 6p21.3 and is part of the *VEGF*/*PDGF* gene family, the cystine-knot superfamily of growth factors. Structurally, VEGF is a 40-kDa heterodimeric glycoprotein. VEGF contains a cystine-knot motif, characterized by disulfide bridges in the protein structure [39]. In humans, VEGF consists of several members: VEGF-A, which has several isoforms, VEGF-B, VEGF-C, VEGF-D, VEGF-E (VEGF virus), placenta growth factor (PlGF), and endothelial-derived VEGF [40].

There are three VEGF receptors: VEGFR-1, VEGFR-2, and VEGFR-3. Neuropilin-1 (NP-1) and neuropilin-2 (NP-2) coreceptors are non-tyrosine kinase receptors, and they selectively attach to certain VEGF subtypes or isoforms. The pro-angiogenic activity of VEGF occurs through the binding and activation of two receptor tyrosine kinases (TKs), which were initially identified as receptors for VEGF-A, namely VEGFR-1 and VEGFR-2. These receptors consist of seven extracellular Ig-like domains, a transmembrane domain, and an intracellular TK domain. Ligand binding induces receptor dimerization and phosphorylation. PlGF binds exclusively to VEGFR-1 with high affinity compared to VEGF-A and VEGF-B, and other family members also specifically bind VEGFR-1 [40].

PlGF was first identified in human placental tissue. It is involved in trophoblast growth and differentiation, trophoblast invasion, and blastocyst implantation. The PlGF gene has four isoforms, PlGF-1 (PlGF131), PlGF-2 (PlGF152), PlGF-3 (PlGF203), and PlGF-4 (PlGF224), which differ in their molecular structure and biological properties. All isoforms have affinity for VEGFR-1. PlGF has no direct mitogenic effect and does not increase vascular permeability, but, under pathological conditions, binds to VEGFR-1, displaces VEGF-A from VEGFR-1, and allows binding of VEGF-A to VEGFR-2, indirectly enhancing the effect of VEGF-A. This increases vascular permeability, cell migration, and proliferation [40].

### KGF

Another growth factor reported to be present in AMSC-MP is KGF [7, 11, 17]. KGF is a monomeric polypeptide measuring 26–28 kDa and is an FGF family member [41]. KGF has been implicated in biological processes such as cell proliferation, development, and differentiation. KGF is encoded by the *FGF7* gene and is made up of 194 amino acids. The human KGF amino acid sequence in UniProtKB (P21781) indicates that this protein contains a signal peptide (residues 1–31) and a KGF chain (residues 32–194), and position 45 is glycosylated during posttranslational modification [41].

KGF has low stability in acidic and neutral pH conditions. Denaturated KGF starts to aggregate at a moderate temperature. The calculated isoelectric point (pI) of KGF is 9.29. The pI is defined as the pH at which the molecule carries no electrical charge or is neutral on average. At a pH below the pI, proteins carry a positive charge, whereas above the pI, they carry a net negative charge [34, 35].

KGF has 19 negatively charged residues, such as Asp and Glu, and 29 positive charge residues, like Arg and Lys. KGF may be unstable under physiological conditions. The instability of the KGF protein is due to the repulsion between the positively charged residues. Therefore, the main cause KGF instability at a neutral pH is thought to be due to its high positive charge and repulsion forces, which lead to protein denaturing and irreversible aggregation [42].

Boroujeni et al. [43] investigated the stability of rhKGF, a truncated form of KGF, under different pH and temperature conditions using molecular dynamics simulation methods. That study showed that the stability of rhKGF increased with a decrease in the total charge at an alkaline pH and low temperature. KGF stability increased significantly at an alkaline pH of 8.5 and 9, presumably due to the presence of a high positive-charge residue. Acidic pH conditions caused instability and aggregation of KGF. In another study, KGF denatured at 400 °C. After a while, the protein began to aggregate, and irreversible particulate and precipitate formation occurred [44].

KGF is involved in various biological processes and has proliferation, antiapoptosis, cytoprotective, epithelial cell movement, and cytoskeletal reorganization effects. Additionally, its mitogenicity is useful for embryonic development, tissue patterning, cell growth, morphogenesis, wound healing, and tissue repair. As a growth factor, KGF acts through a variant of the receptor-2IIIb FGF (FGFR2b), which is expressed by epithelial cells [25]. The FGFR2b receptor is hereafter referred to as the KGF receptor (KGFR). KGFR is part of the FGFR family of receptor TKs, which are activated in the presence of heparin/ HSGAG. The binding of KGF to KGFR requires heparin/HS as a coreceptor. This process includes the dimerization of KGFR and activation of its kinase domain, inducing autophosphorylation of the receptor [45]. To bind to the receptor and its ligands, KGF has a positively charged site called the heparin binding site, and a neutral site that binds to KGFR.

The proliferative effect of KGF occurs due to activation of the RAS and Raf/MAPK/ERK pathways after KGFR dimerization and phosphorylation of the tyrosine kinase domain [46]. Its antiapoptotic effect occurs due to activation of p21-activated kinase 4 (PAK4), which then activates the antiapoptotic-Akt-dependent pathway by recruiting PI3K, which then arranges antiapoptotic genes [47]. Activation of the antiapoptotic pathway by the ERK1/2 pathway reduces the cellular inflammatory response by inducing the expression of the cytoprotective genes nuclear factor erythroid 2-related factor 2 (*NRF2*) and heme oxygenase-1 (*HO1*) in epithelial cells [48, 49].

KGF regulates epithelial cell motility and cytoskeletal reorganization through activation of Src-Cortactin, which phosphorylates paxillin and activates GTPases such as Rho, Rac, and Cdc42, resulting in lamellipodia extension, actin increase, and cell mobility and migration [48]. During mitogenicity, autophosphorylation of the tyrosine kinase domain of KGFR results in the activation of phosphatidylinositol hydrolysis. PLCγ activation hydrolyzes phosphatidylinositol-4,5-diphosphate to inositol-1,4,5-triphosphate and diacylglycerol, which stimulate protein kinase C and increase intracellular Ca^2+^ and subsequent mitogenic activity through upregulation of target genes [50]. Currently, palifermin, a recombinant preparation of human KGF, is a growth factor cocktail used for therapy. For example, a study by Spielberger et al. [51] showed that giving 60 g per kilogram of body weight of palifermin daily reduces the duration and severity of oral mucositis after intensive chemotherapy and radiotherapy for hematological cancer.

Based on the explanation above, it can be seen that various kinds of growth factors present in AMSC-MP, namely bFGF, VEGF, TGF-β, EGF and KGF have their respective functions and activities. In addition, each growth factor is formed by different proteins resulting in molecules with different physicochemical properties and stability. This knowledge will assist in the manufacturing process and application of AMSC-MP as a therapeutic agent.

## Biological activities of AMSC-MP for tissue regeneration

### Wound healing

The wound repair process is divided into four main phases: hemostasis, inflammation, proliferation, and dermal remodeling [52]. Wound repair begins with the hemostasis phase, in which platelet formation blood loss and the initial fibrin matrix begins to form. Platelets are critical in the recruitment of immune cells to wound tissue, either by capturing immune cells directly or by releasing chemokine secretomes. Platelet secretomes also contain growth factors that stimulate resident skin cells, including fibroblasts and keratinocytes [52].

Furthermore, in the inflammation phase, necrotic cells and damaged tissue release damage-associated molecular patterns and resident immune cells, such as mast cells, Langerhans cells, T cells, and macrophages, respond by activating inflammatory pathways. In this phase, proinflammatory cytokine and chemokine release attracts leukocytes in the circulation to the injured tissue. Monocytes already in the wound tissue differentiate into macrophages. Macrophages engulf necrotic cell remnants and pathogenic material [52]. Activation of macrophages is influenced by proinflammatory stimuli, such as lipopolysaccharide and interferon-gamma (IFN-γ), and their activation enhances inflammation by releasing reactive oxygen species (ROS), inflammatory cytokines (e.g., IL-1, IL-6, and TNF-) and growth factors (e.g., VEGF and PDGF).

Macrophages control the degradation of extracellular connective tissue by enzyme secretion and phagocytosis and regulate wound matrix remodeling through the production of growth factors such as PDGF, TGF, ILs, and TNF [53]. These growth factors influence the regrowth process, epithelialization, fibroplasia, and angiogenesis [52, 54]. All three stages occur in the proliferation phase, starting when keratinocytes migrate to close the wound, followed by angiogenesis, and then fibroblasts replace the initial fibrin clot with granulation tissue [55]. Fibroplasia begins about 5 d after injury and continues for 2 weeks. Fibroblasts migrate into the wound and replicate in response to mediators released during inflammation. These mediators include C5a, fibronectin, PDGF, FGF, and TGF61. Remodeling of the extracellular matrix (ECM) spans the entire injury response, beginning with the initial deposition of a fibrin clot and ending several years later with the formation of a mature, type I collagen-rich scar.

Two types of FGF members play an important role in the wound healing process, among which the most important are KGF and bFGF. Both are present in AMSC-MP. Qu et al. (2018) showed that the combination of KGF and bFGF in a collagen delivery system increased cell migration in the wound healing process, accelerating wound closure [56]. bFGF improved wound healing in animal models and clinical studies. KGF is a cytokine that exerts a specific mitogenic effect in epithelial cells. This effect has been reported to be a key factor in wound healing, as it is weakly expressed in human skin but is strongly upregulated after skin injury [56].

The synergistic effect of bFGF and KGF can be observed during the wound healing process. Re-epithelialization begins within hours of injury, and bFGF and KGF promote cell proliferation. KGF stimulates keratinocyte migration, while bFGF promotes fibroblast migration and stimulates the production of collagenase, suggesting that bFGF and KGF have complementary roles in wound healing. Together, bFGF and KGF may also stimulate the accumulation of vascularization-related cells. However, other studies have shown that KGF affects ongoing inflammation and scar formation [57]. These negative effects can be minimized by bGFG as an antiscarring agent [58].

bFGF in wound healing was also investigated by Zhang et al. (2018), in which hydrogels combined with bFGF increased the wound healing process. Hydrogels derived from gum arabic, pectin, and divalent calcium ions help increase the stability of FGFs and provide a sustained release effect [59]. Fibroblast scratch assays showed that the hydrogel FGF formulation could close a wound within 12 h, while in controls, wound closure only started at the 12^th^ hour. In an in vivo study conducted by creating a full-thickness skin incision on the back of mice, the hydrogel treatment group with bFGF showed the fastest wound closure compared to the negative control group [59].

Furthermore, the metabolite products of stem cells have a high potential for wound healing efficacy, based on preclinical and clinical studies on stem cells. The use of human AMSCs for wound healing has also been investigated in vivo in male mice in a heat-induced apoptosis model [60]. The administration of 2 × 10^6^ cells injected subcutaneously into the wounded skin showed accelerated re-epithelialization. Wound closure occurred on day 7, and cytokines, including PAI-1, C-GSF, periostin, and TIMP-1, have been reported to activate the PI3K/AKT pathway, which plays a vital role in epithelial cell and dermal fibroblast migration and proliferation. In another study, the use of human adipose-derived MSCs and placenta-derived MSCs in amniotic membrane grafts accelerated wound healing in Wistar rats with an excisional wound splinting model at day 7 [61]. The processes of re-epithelialization, collagen remodeling, and neovascularization occur more quickly by embedding these cells on the amniotic membrane as wound dressing, and this membrane produces various growth factors such as TGF-α, TGF-β, bFGF, EGF, and KGF, cytokines such as IL-4, IL-6, and IL-8, as well as matrix metalloproteinase inhibitors. A review by Huang et al. (2020), which explored the use of MSCs in preclinical and clinical studies for wound healing, showed that metabolite products play an active role in tissue repair and wound healing [53]. Tissue repair occurs through the stimulation of cell differentiation and paracrine action, involving growth factors such as bFGF, hepatic growth factor (HGF), EGF, KGF, VEGF, and TGF-β, and also cytokines such as IL-10, to reduce inflammation and accelerate the angiogenesis, granulation, re-epithelialization, and wound closure processes.

## Potential uses of AMSC-MP in gastrointestinal injury therapy

In the gastric mucosa, TGF-α controls cell proliferation under normal conditions and after acute injury, while EGF controls cell proliferation during the healing of chronic ulcers. When the gastric mucosa is injured, growth factors predominantly restore the epithelial component, while bFGF and VEGF promote restoration of the connective tissue and angiogenesis in the injured mucosa. Granulated connective tissue, which grows under the stimulation of bFGF and VEGF, is the primary source for regenerating connective tissue lamina propria and microvessels within ulcer scars. Other growth factors such as insulin-like growth factor, KGF, hepatocyte growth factor, and trefoil peptides also act in gastrointestinal (gastric ulcers, colitis) regeneration following injury [62].

Research by Wei et al. (2022) showed that KGF in combination with polydopamine (PDA) and HA nanoparticles successfully prevented abdominal adhesions and promoted the repair of mesothelial cells in the injured peritoneum [63]. More importantly, PDA-KGF NPs combined with HA reduced collagen deposition and fibrosis and inhibited the inflammatory response [63].

KGF function is determined by phosphorylation of the protein tyrosine kinase SRC. When KGF binds to its receptor, SRC is phosphorylated by KGFR. In a study, we evaluated the phosphorylation level of rat Src in the injured peritoneum 7 d after surgery. The levels of phospho-Src protein in rat peritoneal tissue were higher in the PDA-KGF NP treatment group compared to that treated with KGF alone. Thus, the in vivo positive effect of KGF is prolonged when KGF and PDA are administered as PDA-KGF nanogels [63].

KGF is also effective for treating ulcerative colitis. Ying-Zheng et al. (2019) reported that KGF encapsulated into neutrophil-like liposomes (KGF-Neus) effectively restored intestinal morphology and function in ulcerative colitis [64] because the neutrophil membrane vesicle (NEM) associated protein, KGF-Neus, is specifically internalized to the area of inflammation [64].

KGF and its receptors are present in the human fetal gastrointestinal tract, and in vitro stimulation of human fetal enterocytes with KGF results in cellular proliferation. KGF expression is increased in patients who undergo surgery for inflammatory bowel disease and is correlated with the degree of intestinal inflammation. In animal models of colitis, KGF administration reduces the degree of mucosal injury [65]. Recombinant KGF treatment has been studied for use in ulcerative colitis; in a clinical phase II study, recombinant KGF failed to induce remission in ulcerative colitis patients, but the maximal therapeutic dose used may have been too low [66].

Another AMSC-MP component, EGF, is associated with mucosal ulcer disease. Decreased EGF levels are associated with mucosal ulcer disease. Patients with duodenal ulcer disease also have decreased EGF levels. EGF supplementation promotes mucosal repair and regeneration in several conditions. In experiments in pigs, EGF significantly reduced esophageal ulceration, structural formation, and mucosal histological damage associated with sclerotherapy. In rats with gastric ulcers, orogastric EGF administered in combination with sucralfate improved ulcer healing [67]. A small human study showed that treatment with intravenous EGF promoted better gastric ulcer healing compared with the antiulcer treatment cetraxate hydrochloride [67].

## Potential use of AMSC-MP in lung injury treatment

Growth factors are involved in all aspects of lung development. The spatial and temporal distribution of FGF10 in the lung determine the airway branching pattern. Some factors participate in more specific developmental programs, such as VEGF in blood vessel formation and FGF7 in type II alveolar cell differentiation [55]. KGF is a critical growth factor in lung development and is protective after lung injury. KGF is an important growth factor for local resident progenitor epithelial cell repair and for mobilization and enhanced engraftment of cytokeratin 5 circulating epithelial progenitor cells, which contributed to the repair of the proximal airway epithelium in a mouse model of syngeneic tracheal transplantation to the injured proximal airway epithelium [68].

KGF induces epithelial cell proliferation and protects against acute lung injury. Leblond et al. (2007) showed that 1 mg/kg of body weight of KGF given intravenously to rats injected with albumin as an asthma trigger reduced extravascular lung water levels. KGF treatment also reduced the number of inflammatory cells in the bronchoalveolar lavage fluid but not in the bronchial mucosa. KGF reduces allergen-induced changes in epithelial integrity and the expression of the intercellular junction proteins catenin and zonular occludens protein-1 [69].

Consistently, Wang et al. (2020) investigated the effect of KGF on the release of inflammatory-related cytokines by damaged bronchial epithelial cells. Compared with the healthy group, KGF and KGFR expression and apoptosis were significantly increased in asthmatic patients. An in vitro study showed that KGF treatment limited IFN-γ and TNF-α–induced apoptosis by inhibiting apoptotic markers in the TNF signaling pathway. KGF limits the release of TSLP, IL-25, and IL-33 by damaging 16HBE 14o cells. In contrast, KGF promotes intercellular adhesion and wound closure of cultured 16HBE 14o cells through increased expression levels of the intercellular junction proteins ICAM-1, β-catenin, E-cad, and Dsc3. In summary, KGF and KGFR may aid bronchial epithelial cell repair in asthma by inhibiting epithelial cell apoptosis while promoting epithelial cell proliferation and migration [70].

TGF-β is associated with acute lung injury. Research by Kan et al. 2014 showed an increase in the expression of TGF-β in rat serum induced with paraquat, a compound that irritates the lungs [71]. *TGF-β1* mRNA expression in rat lungs was also significantly increased. Many inflammatory cells were observed infiltrating the alveoli of the injured lungs. The abnormal expression of *TGF-β1* was hypothesized to be important in the pathogenesis of chronic inflammatory and immune lung diseases, including asthma, chronic obstructive pulmonary disease, and pulmonary fibrosis [72]. In the future, cytokines and their inhibitors may provide new therapies for treating acute lung injury and pulmonary fibrosis.

Heparin-binding EGF-like growth factor (HB-EGF) reduces inflammation, maintains intestinal barrier function, and protects the lung from acute injury in several models of intestinal injury. Another study investigated the impact of HB-EGF by comparing burn-treated mice (25% of total body surface area) with burn-infected mice after two enteral doses of HB-EGF (1200 mg/kg/dose) [73]. The control mice had increased pulmonary myeloperoxidase levels, lung and spleen apoptosis, airway resistance and bronchial reactivity, and intestinal permeability. These effects were significantly reduced in burn-injured mice treated with enteral HB-EGF [73].

FGF2 is closely involved in endothelial cell migration, proliferation, and injury repair. Recombinant FGF2 was injected peritoneally at a 0.1 mg/kg dose in septic mice induced by ligation and cecal puncture. FGF2 treatment reduced the inflammatory response, attenuated pulmonary capillary leakage, reduced lung injury, and increased survival in septic mice. Endothelial injury and macrophage inflammation induced by LPSs are inhibited by FGF2 administration via the AKT/P38/NF-κB signaling pathway [74].

## Potential use of AMSC-MP in bladder and renal injuries

Surgical and traumatic injuries to the bladder initiate a complex series of biological processes that result in wound healing. This involves cellular proliferation, migration, and differentiation; removal of damaged tissue; and production of extracellular matrix, all of which may be controlled by growth factors. KGF is induced in the skin following incisional injury. During the early phases of bladder wound healing, mRNA levels of *KGF* and *TGF-α* increased, and exogenous KGF directly affected urothelial proliferation [75].

Among the growth factors that affect the bladder are KGF and FGFs; KGF increases cyclophosphamide-induced bladder injury. Cyclophosphamide is often used to treat cancer and rheumatic and kidney diseases. Acrolein, its metabolite, is a toxic metabolite concentrated in urine that can cause acute hemorrhagic cystitis (7%–45% incindence rate) and urothelial cancer (4%–15% incidence rate, depending on the dose). In cyclophosphamide-induced urothelial injury, increased apoptosis of intermediate and basal cells was observed. KGF prevented apoptosis of deeper urothelial cells (UPK3 + intermediate and KRT5 + intermediate/basal cells), likely via activation of AKT [76].

Evidence suggests that KGF regulates bladder cell development and function and is directly responsible for urothelium proliferation. In a study by Narla et al. (2020), mice were given a 5 mg/kg injection of KGF dissolved in PBS 24 h before cyclophosphamide injection and showed increased urothelial regeneration compared to controls [76]. KGF pretreatment blocked cyclophosphamide-induced intermediate and basal cell apoptosis, possibly via phosphorylated AKT, and promoted ERK-mediated phosphorylated KRT5 + /KRT14 − cell proliferation, leading to urothelial regeneration. The effect of KGF on bladder injury was also found in a study by Jaal et al. (2007) [77], in which there was an increased positive response on day 2 in 50% of mice after a single injection of palifermin at a dose of 15 mg/kg [77].

In addition to KGF, bFGF also affects bladder injury. Chen et al. (2010) explored the ameliorative effect of collagen-based bFGF for bladder regeneration in a mouse model [78]. A bladder with a subtotal cystectomy was grafted with collagen membranes coupled with 0.56 nmol of CBD-bFGF. As a result, collagen/bFGF mice had faster collagen scaffold degradation and better bladder wall cell growth but no bladder stone formation [78].

In addition to bladder injury, FGF positively affects acute kidney disease. Zhou et al. investigated the effects of FGF2 in acute kidney disease using Sprague–Dawley and NRK-52E cells [79]. FGF2 significantly increased tissue apoptosis in acute kidney disease by inhibiting excessive ER stress. Moreover, FGF2 also reduced ER overstress and apoptosis in cultured NRK-52E cells injured with tert-butyl hydroperoxide [80].

## Potential uses of AMSC-MP for bone regeneration

The bone response to injury begins with an inflammatory phase. Bleeding from the fracture-surrounding soft tissue results in forming a fibrin clot and fracture hematoma. Subsequently, inflammatory cytokines are released, inducing angiogenesis and mesenchymal progenitor cell proliferation. These mesenchymal progenitors rapidly proliferate, forming an initial soft callus. The soft or primary callus response occurs within two weeks of injury. The degree of callus formation is proportional to the degree of motion at the fracture gap [81].

In the second stage of repair, the necrotic bone ends undergo resorption, and the mesenchymal progenitor cells proliferating at the injury site begin to differentiate into chondrocytes to form a cartilaginous callus and osteoblasts for intramembranous bone formation at the fracture margins. The mechanisms that control the influx, proliferation, and differentiation capacity of mesenchymal progenitor cells are critical components of the fracture healing process. For instance, BMP and Wnt signaling play roles in mesenchymal cell differentiation into osteoblasts and chondrocytes [81].

During the establishment and maturation of the soft callus, growth factors (e.g., TGF-β, PDGF, GDF-5, FGF-1, and IGF-II) and hormones (e.g., PTHrP) are involved in the recruitment and proliferation of fibroblasts and MSCs. They also play an essential role in inducing MSC differentiation into osteoblasts or chondrocytes [82]. Once chondrocytes form, endochondral ossification occurs, and a hard callus is formed via woven bone in the third stage of healing. Whether this endochondral bone formation process is equivalent to that which occurs during bone growth is not completely understood [83]. The use of human AMSC in bone tissue regeneration has been reported in several studies, including in in vitro and in vivo studies and clinical trials, as summarized by Li et al. (2020) [84]. AMSC treatment in models of collagen-induced arthritis, intervertebral disc degeneration, rheumatoid arthritis, and osteoarthritis has shown anti-inflammatory, angiogenic, and immunomodulatory effects, all of which play important roles in tissue remodeling. Several growth factors and cytokines present in human AMSC play an important role in the regeneration process of bone defects. These include HGF, FGF7, BMP-2, VEGF, IL-6, and IL-8. Clinical trials in patients with bone defects of human AMSC administration either by subcutaneous injection or implantation into the hypodermis with a polymer or scaffold have shown increased proliferation, and osteoblastic differentiation of BMSCs increased osteogenesis and endogenous bone regeneration.

Itoh et al. (2007) confirmed that FGF/FGFR signaling plays a role in osteogenesis. FGF/FGFR signaling does not directly induce osteoblast differentiation but modulates it. FGF2 and FGF9 likely induce the proliferation of osteoblast cell lineages and the induction of angiogenesis, and FGF18 promotes osteoblast differentiation [85].

Wilkie et al. (2005) published an in vitro analysis of bone marrow-derived MSCs in which FGF18 enhanced osteoblast differentiation by activating FGFR1 or FGFR2 signaling [86]. Additionally, overexpression of FGF18 by lentiviral infection or direct addition of FGF18 to culture media induced the expression of osteoblast marker genes in C3H10T1/2 fibroblastic cells. Treatment with FGF18 in mouse-derived MSCs under differentiation-inducing conditions showed increased expression of osteoblast differentiation markers and mineralization [86].

Low-dose FGF18 treatment with osteogenic induction of bone morphogenetic protein 2 (BMP2)-dependent bone protein from MC3T3-E1 cells increased mineralization, whereas high-dose treatment inhibited the process. Additionally, FGF18-soaked heparin-coated acrylic beads accelerated osteoblast differentiation in mouse fetuses by regulating BMP2 expression in 90 osteoblast cell lineage cells [87]. FGF2 stimulates mitosis and cell proliferation, including of fibroblast and endothelial cells, which plays a vital role in maintaining these cells in tissue repair processes [88]. On the other hand, FGF18 stimulates cellular osteogenesis through the upregulation of bone morphogenetic proteins.

## Potential uses for skin rejuvenation in skin aging

Together with other growth factors and cytokines, EGF directly affects collagen, elastin, and ECM biosynthesis, but its binding and signaling diminish with age. Aged cells in the skin produce ROS, and the mitochondria of these cells disrupt tissue complexes by cleaving membrane-bound receptors, ECM proteins, growth factors, and other signaling ligands in the dermal microenvironment [89]. Reduced EGF binding and signaling with age can cause collagen degradation in the skin [90, 91]. The rapid degradation of collagen in the skin leads to loss of elasticity and the appearance of skin wrinkling [92]. Decreased expression of EGFR also occurs in aging dermal fibroblasts in the ECM, is associated with reduced cell migration and proliferation, and ultimately leads to skin flexibility and elasticity loss. EGF helps reduce the effects of aging by supporting skin regeneration by stimulating cell renewal through the interaction of keratinocytes and fibroblasts. EGF plays an important role in forming fibroblasts in the dermis by stimulating collagen production via activation of EGFR [89]. The topical use of growth factors is a safe and effective medical treatment [93]. Applying EGF to aging skin can increase fibroblast proliferation [91]. Thus, EGF is a potential therapeutic antiaging agent for the skin.

A clinical trial of human umbilical cord-derived MSC-conditioned media administered by microneedle resulted in good efficacy as an antiaging product and provided an excellent potential for skin rejuvenation [94]. That study reported that the tested conditioned media contained growth factors, including EGF, VEGF-A, VEGF-D, HGF, FGF-2, and others. Furthermore, the administration of MSCs reduced the melanin index and brown spots on the skin. Additionally, wrinkles and skin pores were reduced, and there was an increase in skin elasticity, indicating an improvement in facial skin texture. Another study showed that administering amniotic fluid MSC-derived conditioned media with microneedles to the face improved the skin texture and reduced wrinkles [95]. Moreover, histologically, there was an increase in the number of dermal collagen bundles arranged more regularly, elastic fibers, and epidermis thickening.

AMSC-MP possess various growth factor and cytokines that enables them to become the agent therapeutics of many therapy.

## Future prospects for AMSCMP for tissue engineering

Many reports have demonstrated the potential use of AMSC-MP for tissue regeneration to improve the appearance of facial skin, in bone regeneration, and in tissue or organ repair. Preclinical reports, including in vitro and in vivo studies, have shown that growth factors and cytokines play an essential role in the tissue repair process, both through stimulation of cell differentiation and proliferation and via an indirect effect on regeneration, including antiinflammatory and angiogenic effects. Clinical trials using conditioned media and MSCs have also reported potent activity for tissue regeneration.

However, protein delivery systems of the cytokines and growth factors present in AMSC-MP still have many shortcomings. Oral delivery is generally preferred, but oral delivery is not a viable method for proteins due to their poor absorption and degradation in the gastrointestinal tract and liver [96]. Oral administration of protein drugs leads to very low bioavailability [97]. Thus, the development of protein formulations is needed to overcome the low permeability of large molecules, the lack of lipophilicity, and rapid inactivation or enzymatic degradation in the gastrointestinal tract as well as protein physicochemical properties that are limiting [97]. For topical uses, growth factors have molecular weights higher than 500 Da, which makes the penetration of the stratum corneum difficult [91]. The ideal characteristics of a substance for a topical delivery system include a relatively low molecular weight (< 500 Da), a low melting point (< 200 °C), moderate lipophilicity (log P 1–3), and high water solubility (> 1 mg/mL), as well as high pharmacological potential [98]. Therefore, a delivery system is needed to help these molecules penetrate the dermis.

The use of AMSC-MP is also limited due to its sensitivity to environmental factors such as temperature, pH, and reactivity during reconstitution. Because of this, the delivery system must optimize the growth factor dose, route of administration, and release kinetics for the safe and effective use of growth factors [99]. For example, KGF has poor in vivo bioactivity. KGF has a short biological half-life and poor stability, its biological activity is susceptible to environmental factors, and it cannot maintain bioactivity for a long time in the presence of other enzymes [45].

In addition to stability, using AMSC-MP for tissue regeneration therapy requires a carrier system capable of local delivery with the controlled release of growth factors. The uncontrolled release of growth factors can cause side effects. Using biomaterials as delivery systems is the most successful strategy for controlled delivery, and they have been developed into various commercially available systems [100]. Based on the explanation above, it can be seen that AMSC-MP has been widely studied for its benefits in various diseases. Thus AMSC-MP has potential as a tissue regeneration therapeutic agent.

## Manufacturing of AMSC-MP for therapeutic products

Presently, autologous cell therapy is primarily used for stem cell-based therapy. However, the small individual scale, insufficient reagents, and inefficient manufacturing process result in an expensive product. The proper indication for use and timing, adequate dosage, and appropriate route of administration still need to be determined for widespread use.

The possible scaling up of AMSC-MP would represent a more efficient means of manufacturing mass-scale products to induce tissue regeneration of greater suitability to community needs rather than individual therapy. Moreover, it would reduce the possibility of immune system rejection of cell therapy. The process of manufacturing AMSC-MP from AMSCs is divided into at least three main stages: isolation of mesenchymal cells from placental tissue, cell culture and incubation, and the harvesting and purification of the metabolite products of cell cultures. Quality assurance involving validation needs to be carried out to identify a sustainable production process and guarantee the quality of AMSC-MP. The validation process includes cell culturing, cell stock storage, the harvesting of conditioned media containing cell metabolites, and their processing into AMSC-MP as bioactive materials. AMSC-MP standardization will then be required to ensure consistent and reliable product quality. Quality parameters such as physicochemical characteristics and growth factor content would constitute important specification parameters of bioactive materials. Through validation, mass-production is possible using tailor-made cell lines under controlled laboratory conditions which provide a high-quality source of bioactive factors necessary to produce mass products efficiently and safely.

Furthermore, large-scale manufacturing should be developed to minimize costs and provide affordable therapeutic products for the public; however, the standardization of AMSC-MP into biological products has substantial challenges. Process validation, quality control, and standardized protocols for isolating, culturing, and cultivating AMSC-MP are required, and these are major challenges for manufacturers. Moreover, regulations that support clinical use are also being developed, so AMSC-MP mass production remains under development. Despite these challenges, developing AMSC-MP-based therapeutic products is worthwhile for providing affordable advanced biological therapeutics for clinical practice.

## Conclusions

AMSC-MP has excellent potential for use in tissue regeneration therapy since it contains a variety of growth factors that provide better efficacy than single growth factors or cytokines alone. In vitro and in vivo preclinical studies have shown that AMSC-MP has biological activities related to wound healing, the repair of bone defects and other bone diseases, tissue repair of damaged organs, including the lungs, bladder, kidneys, and in the gastrointestinal tract, as well as skin rejuvenation related to antiaging effects, providing excellent efficacy and demonstrating a good safety profile. Some reports have shown satisfactory results in clinical trials in patients with certain diseases. There is a high clinical demand for AMSC-MP as an alternative biological therapy, but further development is needed regarding its stability and the identification of a delivery system to provide maximum efficacy. Additionally, product development from the laboratory to a mass production scale requires further effort. Moreover, quality assurance is needed for biological product materials and complex manufacturing processes, which is the main challenge that must be addressed to optimize the use of AMSC-MP as a therapeutic agent.

## Data Availability

Not applicable.

## Abbreviations

AMSC: Amniotic Membrane Stem Cell
AMSC-MP: Amniotic Membrane Stem Cell Metabolite Products
BAL: Bronchoalveolar lavage
BMP: Bone morphogenic protein
EGF: Epidermal growth factor
ELISA: Enzyme-linked immunosorbent assay
FD: Freeze-dried
GDF: Growth differentiation factors
HA: Hyaluronic acid
HGF: Hepatic growth factor
IGF: Insulin-like growth factor-I
ILD: Interstitial lung diseases
KGF: Keratinocyte growth factor
KGFR: KGF receptor
MAPK: Mitogen-activated protein kinase
MSC: Mesenchymal stem cells
PDGF: Platelet-derived growth factor
PlGF: Placenta growth factor
ROS: Reactive oxygen species
TGF: Transforming growth factor
TK: Tyrosine kinases
VEGF: Vascular endothelial growth factor
